# Transgenerational effects of maternal diet on metabolic and reproductive ageing

**DOI:** 10.1007/s00335-016-9631-1

**Published:** 2016-04-25

**Authors:** Catherine E. Aiken, Jane L. Tarry-Adkins, Susan E. Ozanne

**Affiliations:** University of Cambridge Metabolic Research Laboratories and MRC Metabolic Diseases Unit, Institute of Metabolic Science, Addenbrooke’s Treatment Centre, Addenbrooke’s Hospital, Cambridge, CB2 0QQ UK; Department of Obstetrics and Gynaecology, The Rosie Hospital and NIHR Cambridge Comprehensive Biomedical Research Centre, University of Cambridge, Box 223, Cambridge, CB2 0SW UK

## Abstract

The early-life environment, in particular maternal diet during pregnancy, influences a wide range of organs and systems in adult offspring. Mounting evidence suggests that developmental programming can also influence health and disease in grand-offspring. Transgenerational effects can be defined as those persisting into an F2 generation, where the F0 mother experiences suboptimal diet during her pregnancy. In this review, we critically examine evidence for transgenerational developmental programming effects in human populations, focusing on metabolic and reproductive outcomes. We discuss evidence from historical cohorts suggesting that grandchildren of women exposed to famine and other dietary alterations during pregnancy may experience increased rates of later health complications than their control counterparts. The methodological difficulties with transgenerational studies in human cohorts are explored. In particular, the problems with assessing reproductive outcomes in human populations are discussed. In light of the relative paucity of evidence available from human cohorts, we consider key insights from transgenerational experimental animal models of developmental programming by maternal diet; data are drawn from a range of rodent models, as well as the guinea-pig and the sheep. The evidence for different potential mechanisms of transgenerational inheritance or re-propagation of developmental programming effects is evaluated. Transgenerational effects could be transmitted through methylation of the gametes via the paternal and maternal lineage, as well as other possible mechanisms via the maternal lineage. Finally, future directions for exploring these underlying mechanisms further are proposed, including utilizing large, well-characterized, prospective pregnancy cohorts that include biobanks, which have been established in various populations during the last few decades.

## Introduction

It is increasingly accepted that the environment during early development has a profound and lasting effect on many aspects of physiology and metabolism later in life. A large body of literature has gradually accumulated demonstrating such effects across generations in both human populations and animal models (Aiken and Ozanne 2014; Padmanabhan et al. 2016; Pembrey et al. 2014). A lasting influence of the early-life environment has been shown on diverse aspects of health including behavioural traits (Constantinof et al. 2015), immunological function (Ingvorsen et al. 2015) as well as cardiovascular and metabolic health (Giussani and Davidge 2013; Reynolds et al. 2015). As the adverse later-life effects of poor maternal diet on the health of adult offspring become clearer, attention has turned to better delineating the molecular mechanisms responsible (Ozanne 2015), and hence developing interventions that could off-set the effects of poor early nutrition (Tarry-Adkins et al. 2014; Zambrano et al. 2016). For the large number of vulnerable individuals who have been exposed to a suboptimal intrauterine environment, a clear understanding of the risks this poses to their own health as well as that of future generations would be highly beneficial. Furthermore, understanding the mechanisms of transgenerational propagation of developmental programming phenotypes is key to finding ways to reverse or ameliorate programming effects.

 Adverse early-life environments are wide-ranging, and a diverse range of stimuli has been evaluated for potential to induce developmental programming phenotypes in offspring. In human populations, these have included exposure to famine (de Rooij et al. 2015; Li et al. 2015), natural disasters (Laplante et al. 2008), traumatic life events (Khashan et al. 2011) and maternal psychological stress (Entringer et al. 2010) during pregnancy. In animal models, alteration of maternal diet (Ong and Ozanne 2015; Penfold and Ozanne 2015), experimentally induced hypoxia (Giussani and Davidge 2013) and micronutrient deficiencies (Mathias et al. 2014) have been found to cause programming effects. However, by far the most frequently studied programming intervention in models is alteration of maternal diet, which is also a common naturally occurring developmental programming stimulus in human populations. In both developed and developing countries, optimizing maternal diet is a challenging public health problem (Kaiser et al. 2014). Globally, rates of obesity among women of childbearing age are rising (Ng et al. 2014a), and as yet the full implications for future generations following these pregnancies are only partially understood.

Many of the most frequently studied phenotypes in both human populations and animal models of developmental programming are those related to offspring metabolic dysfunction. In particular, obesity and insulin sensitivity have been the later-life endpoints in many seminal developmental programming studies, both in immediate first-generation offspring (Mahmood et al. 2013; Segovia et al. 2015) and across generations (Jimenez-Chillaron et al. 2015; Masuyama et al. 2015; Zambrano et al. 2005). We consider how these parameters may be affected by grand-maternal diet and the contribution of accelerated cellular ageing as a key mechanism driving these outcomes.

Female reproductive function is intimately interconnected with metabolism in adulthood (Michalakis et al. 2013; Padmanabhan and Veiga-Lopez 2014), and it is becoming increasingly clear that reproductive function in female offspring is highly sensitive to the influence of the early-life environment (Sloboda et al. 2011; Zambrano et al. 2014). Particularly in the context of studying cellular ageing as a feature of transgenerational developmental programming, female reproductive function is a useful model because of the shorter time-scale of reproductive than somatic ageing (Albertini 2012; Broekmans et al. 2009; Li et al. 2012). The rapidity of reproductive senescence compared to ageing in other tissues cumulates in the loss of reproductive capacity in the adult female long before overt signs of ageing would be expected in other organ systems. This shortened time-span is reflected in the early reduction in telomere length in reproductive tract tissues compared to other tissues in rat first-generation offspring exposed to a low-protein maternal diet during pregnancy (Aiken et al. 2013). Most recently, new studies have indicated that reproductive dysfunction may also persist across generations (Aiken et al. 2015), and hence this is an instructive outcome to study alongside metabolic disturbance. There is a wide range of parameters that can be used as surrogates of female reproductive age at any given life-stage. Many of these, such as measurement of total primordial follicular reserve are feasible in animal models but not in human populations. In human populations, anti-Mullerian hormone (AMH) is often measured as a surrogate marker of antral follicular reserve, but this has not yet been performed across subsequent generations in human studies. We thus derive indirect evidence about female reproductive ageing in transgenerational human cohorts from what scanty evidence is available on quantifiable clinical outcomes instead.

### Defining transgenerational effects

The definition of the term ‘transgenerational’ is often unclear in the developmental programming literature, but in this review it is used specifically to denote an effect that persists at least into an F2 generation after initial dietary stimulus during an F0 pregnancy (also referred to as an intergenerational effect). It has been argued elsewhere that any developmental programming effect may be considered transgenerational by definition, as an intervention in the F0 generation (usually during pregnancy) influences the outcome for the next generation (F1) (Pembrey et al. 2014). However, this definition includes the direct influence of maternal intervention on the foetus in utero, and does not require postulation of a mechanism by which developmental programming effects are recapitulated in a subsequent generation. Conversely, the most conservative definition of transgenerational effects are those that persist into an F3 generation, as the germline cells that become the F2 generation are physically present during the later stages of F0 gestation and thus could theoretically be directly affected by the original intervention (Skinner 2008). However, effects that persist into the F3 generation and are mediated by maternal diet in the F0 pregnancy have not been widely studied and hence are less informative to consider. This is an important area for future work. By contrast, a number of studies have shown that there are adverse effects of maternal diet that persist in grand-offspring (F2 generation) [reviewed in (Aiken and Ozanne 2014)]. In this review, we designate the grandparental generation subjected to the dietary stimulus as F0, the first generation of offspring as F1 and the grand-offspring (second generation) as F2.

### Transgenerational developmental programming in human populations

#### Transgenerational metabolic programming in human cohorts

Few studies have examined transgenerational developmental programming effects in human cohorts, for several important methodological reasons.

The first limitation in human studies is that maternal diets are wide-ranging between and within populations, as well as at an individual level. The problems with retrospective food recall in human populations of any sort have been extensively documented elsewhere (Ishihara 2015). In pregnant populations, food records many present extra challenges regarding validity and interpretation (Barbieri et al. 2015). The generation times in human populations are long, and usually preclude the easy set-up of prospective transgenerational cohorts that will have sufficient follow-up rates in order to draw firm conclusions.

The second major limitation in human studies is the measurement of outcomes attributable to maternal diet in the F0 pregnancy. Both the length of generation times and the complexity of human experience between study time points mean that linking offspring and particularly grand-offspring outcomes back to exposures in the index pregnancy is fraught with difficulty. The methodological difficulties inherent in teasing out an effect of maternal diet during pregnancy in generations beyond F1 make such studies highly complex, even when good retrospective data are readily available. Hence there are few studies that have attempted to distinguish the effect of maternal diet in transgenerational effects in human cohorts (Table 1), and those that do are mainly limited to immediate postnatal outcomes in the second generation, particularly birthweight (Alwasel et al. 2013; Rickard et al. 2012; Stein and Lumey 2000).

**Table 1:** Key human epidemiological studies reporting metabolic outcomes in second-generation (F2) offspring

Study	Population	F2 generation outcome
Immediate postnatal outcomes only in F2 generations		
Alwasel et al. (2013)	F2 offspring whose mothers were in utero during Ramadan (Tunisia)	Decreased birthweight Lower ponderal index Lower placental weight
Stein and Lumey (2000)	F2 offspring whose mothers were exposed to the Dutch hunger winter in utero (Netherlands)	Decreased birthweight
Rickard et al. (2012)	F2 offspring whose mothers were in utero during seasons of poor nutrition (Gambia)	Variable effects on birthweight, depending on years of study
Early adulthood outcomes ± immediate postnatal outcomes in F2 generation		
Bygren et al. (2014)	F2 offspring whose grandparents underwent sharp changes in their food intake between consecutive years (Sweden)	Increased risk of cardiovascular mortality in female F2 offspring (dependent on F0 paternal grandmother’s food intake—no effect with any other grandparent)
Painter et al. (2008)	F2 offspring of F1 parents who were in utero during the Dutch Hunger, compared to offspring whose parents were in utero before or after the famine. F2 outcomes reported in interviews with F1 study participants (Netherlands)	Decreased birth length and increased ponderal index No effect on birthweight Increased incidence of poor health later in life
Veenendaal et al. (2013)	F2 offspring of F1 parents who were in utero during the Dutch Hunger, compared to offspring whose parents were in utero before or after the famine. (Netherlands)	Higher adult body weight (F1 father exposed only) Higher BMI (F1 father exposed only)
Li et al. (2015)	F2 offspring of parents who were in utero during Chinese famine 1959–1962 (China)	No effect on cognitive function in adulthood

Studies of transgenerational developmental programming effects in human cohorts have thus had to make use of a variety of natural experiments. A key example is from an isolated Swedish community (Oberkalix), for which historical records are available that allow dietary intake data to be reconstructed over generations. Examination of the records shows that where the paternal grandmother (F0) experienced dramatic changes in food availability during her own growth, her female grandchildren (F2) were at higher risk of cardiovascular mortality (Bygren et al. 2014). No effect of the maternal grandmother’s diet on cardiovascular risk in the grandchildren was discernable within the study. This implies that epigenetic transmission via the spermatozoa might be the mechanism by which cardiovascular risk is passed from the F1 to the F2 generation in this population. However, there are no data available on the transmission of metabolic or fertility-associated outcomes as yet from this population. Similarly, the grandchildren of women who were pregnant during the Dutch hunger winter (November 1944–May 1945) have been studied for evidence of second-generational effects. The first-generation offspring of this cohort were among the earliest paradigmatic examples of human developmental programming (Roseboom et al. 1999), and showed clear evidence of metabolic dysfunction (Lumey et al. 2009; Ravelli et al. 1999). The adult grandchildren (F2 generation) had more reported episodes of poor health from any cause (Painter et al. 2008). Although there was no evidence that grand-maternal exposure to famine during pregnancy resulted in an increased incidence of either cardiovascular or metabolic disease, at the time of the study the F2 generation were in their early thirties, and thus would not be expected to have developed clinical manifestations of these problems as yet. More recently, it has been shown that adult grand-offspring whose fathers were exposed to famine in utero had higher BMI than a control population, but this was not true where the famine exposure was via the maternal line (Veenendaal et al. 2013). Other human populations exist in which studies of the transgenerational impact of intrauterine under-nutrition have been undertaken, but as yet no studies of the metabolic or reproductive outcomes in offspring have been reported. These include famine exposure in China during 1959–1963, in which second-generation cognitive functioning was found to be unimpaired (Li et al. 2015). A further example comes from the study of children whose grandmothers fasted for Ramadan during pregnancy (Alwasel et al. 2013). In this study, the grandchildren were lighter at birth and had lower placental weights; this raises the possibility of later-life metabolic effects, and future study of these children will be of great interest.

#### Transgenerational reproductive programming in human cohorts

Evidence for developmental programming effects on female reproductive parameters from human cohorts is extremely limited. The more general methodological limitations for linking maternal diet with health and disease in children and grandchildren are applicable to these studies, but there are also added complications specifically connected with study of reproductive function in human populations. These include social, behavioural and cultural determinants of childbearing and family structure, which can easily mask the biological aspects of female fecundity. Trends towards widespread contraceptive use, small family size and delayed childbearing in many populations around the world mean that developmentally determined problems with conceiving and carrying pregnancies are difficult to disentangle. Thus, there are very few studies that have attempted study of reproductive outcomes following exposure to suboptimal maternal diet. Accepting these limitations, the reproductive outcomes of adult females who were in utero during the Dutch hunger winter have been studied (Yarde et al. 2013). There was no discernable effect of famine exposure on fertility or pregnancy outcomes; however, there was an increased likelihood (24 % increase) that a woman who had been exposed to famine in utero would undergo menopause at any given age (Yarde et al. 2013). There are, as yet, no studies in which the reproductive outcomes of grandchildren following suboptimal maternal diet during pregnancy have been examined.

### Insights from animal models

Although human studies have established the principle that metabolic and reproductive effects can be transmitted across generations, the study designs that are feasible in existing cohorts have not yet been sophisticated enough to provide very detailed mechanistic insights into how transgenerational developmental programming might occur. Thus in order to delve further into the phenotypes described, it is necessary to examine what information regarding mechanisms can be gleaned from animal models of maternal diet-induced transgenerational developmental programming. Such models have primarily been rodent-based, and include a wide range of maternal dietary interventions including low-protein diet (Aiken et al. 2015; Zambrano et al. 2005), low calorie diet (Martinez et al. 2014; Radford et al. 2014; Thamotharan et al. 2007) and high-fat or obesogenic diets (Dunn and Bale 2011; Gniuli et al. 2008; Pentinat et al. 2010). In addition, transgenerational programming effects have been demonstrated in other species, notably with glucocorticoid exposure in the guinea-pig (Crudo et al. 2012; Iqbal et al. 2012; Long et al. 2013) and obesogenic maternal diet in the sheep (Shasa et al. 2015).

The most frequently suggested mechanisms through which developmental programming effects may occur include (i) structural effects on tissues and organs, (ii) epigenetic programming of gene expression, (iii) glucocorticoid effects or (iv) accelerated cellular ageing. Evidence for maternal diet affecting the metabolic and reproductive outcomes of grand-offspring has been demonstrated via all three suggested mechanisms (Aiken et al. 2015; Radford et al. 2014; Thamotharan et al. 2007).

#### Structural effects

Maternal energy restriction in a rat model has been shown to lead to reduced pancreatic beta cell mass at birth in both the F1 and F2 generations (Blondeau et al. 2002). Similar reductions in beta cell mass have been observed in the F2 generation in a mouse model after exposure to low-protein diet during F0 pregnancy (Frantz et al. 2011). More recently, it has been shown that a grand-maternal (F0) high-fat diet in the mouse has the opposite effect, with an increase in pancreatic mass and islet density persisting to the F2 generation (Graus-Nunes et al. 2015). In a mouse model of grand-maternal vitamin D deficiency, not only the size of pancreatic islets, but also the amount of steatosis present in the liver of the F2 generation was affected by the transgenerational exposure (Nascimento et al. 2013). However, in order to have a direct effect on the structure and function of organs in the F2 generation, it is not sufficient for the F1 generation merely to have structural alterations in organs such as the pancreas and liver. For a structural effect to be directly responsible for transgenerational developmental programming effects, it would need to be present in the female reproductive tract. Such an example could involve programming in the F1 generation affecting the vascular supply to the uterus (Hemmings et al. 2005), such that the F2 generation developing within the altered intrauterine environment was subjected to an effective nutrient restriction, despite a normal maternal diet during F1 pregnancy (Leese et al. 2008). The somatic ovary is also known to be a target for modulation by developmental programming interventions (Aiken et al. 2013; Bernal et al. 2010). Structural anomalies in F1 offspring may also be connected with adverse outcomes in the F2 generation via indirect mechanisms, if they impair the maternal adaptations to normal pregnancy. Such examples include developmental of gestational diabetes due to impaired maternal glucose tolerance that could be related to structural changes in the F1 pancreas (Aerts et al. 1997) or where maternal nephron number is reduced leading to impaired renal function and subsequent hypertension or pre-eclampsia in pregnancy.

#### Epigenetic programming of gene expression

Transcriptional programming of gene expression, most often assayed by methylation status, is one of the most widely studied outcomes in developmental programming models. Methylation patterns as a means of transmission of developmental programming effects are often viewed as problematic, due to the concept that DNA methylation patterns are reset both in the primordial germ cell and the early embryo (Seisenberger et al. 2012). However, recent evidence suggests that this ‘wiping’ of methylation may not be complete, and that at least some methylation can be directly inherited (Borgel et al. 2010). There is clear evidence that while the methylation status of the genome can be affected by calorie restriction during F0 pregnancy, that imprinted genes are no more susceptible to such perturbation than is the rest of the genome (Radford et al. 2012). In keeping with this idea, methylation status of the promoter regions in key genes regulating metabolism is altered in response to high-energy diet through to the F3 generation in the mouse (Burdge et al. 2011). Convincing evidence for the role of epigenetic modification of the gametes comes from observed transgenerational developmental programming effects solely via the paternal lineage (Fullston et al. 2012; Martinez et al. 2014; Radford et al. 2014). These studies show that male gametes can acquire stable epigenetic alterations from paternal dietary manipulations, which are sufficient to induce adverse metabolic phenotypes across multiple generations of offspring (Fullston et al. 2012). An equivalent effect has not been seen for transgenerational transmission via the female line, where the role of the gamete is more complex, as it contributes not only the female pronucleus but also the ooplasm to the developing conceptus. The ooplasm comprises the substance of the embryo through early cell divisions until cleavage has ceased, after the point of implantation in the mammal (Aiken et al. 2008). Modifications to the ooplasm, in particular the mitochondria and mitochondrial DNA, which is so abundant within the oocyte (Aiken et al. 2008), could provide a further mechanism for gamete involvement in transgenerational developing programming via the maternal lineage (Zander-Fox et al. 2015). There is evidence that the ooplasm itself is susceptible to maternal diet effects from a mouse model of a high-fat diet, which induced altered mitochondrial activity in the oocyte that was transmitted directly to the developing zygote (Igosheva et al. 2010).

Although modification of DNA methylation patterns is the most widely studied, there are a number of other possible mechanisms of epigenetic modification that may contribute to transgenerational developmental programming. These include histone modification and differential expression of miRNAs. Debate is ongoing about the stability of histone modifications across generations in order to provide a mechanism for transgenerational inheritance of developmental programming effects, but there are some indications that histone marks may be stably inherited (Gaydos et al. 2014). While links between maternal diet and adverse metabolic outcomes in F1 offspring have been observed via histone modification (Strakovsky et al. 2014), there is yet to be sufficient evidence from animal models that they can lead to transgenerational developmental programming effects. Micro RNA expression is altered by maternal diet in a number of animal models (Fernandez-Twinn et al. 2014). Expression of miRNA in sperm may be altered by high-fat feeding via the paternal line (Fullston et al. 2013), demonstrating the potential for transgenerational programming effects via this route. Interestingly, in a mouse model of maternal separation causing altered miRNA expression in the sperm and subsequent impairment of glucose homeostasis in the offspring, the transfer of these purified miRNAs to the oocyte could recapitulate some of the metabolic effects in the offspring even in the absence of any initial stressor (Gapp et al. 2014). This goes some way towards establishing miRNA manipulation as a potential causative mechanism of transgenerational developmental programming effects.

#### Glucocorticoid effects

Prenatal exposure to glucocorticoids, while not strictly a maternal dietary modulation, is nonetheless a commonly modified aspect of the early-life environment in both human pregnancy and animal models (Crudo et al. 2012; Long et al. 2013), which is informative to consider. Additional glucocorticoid exposure can occur as a result of synthetic hormone administration, or endogenously as a result of maternal stress. It has been demonstrated in the guinea-pig that exogenous glucocorticoid administration during an index F0 pregnancy can alter DNA methylation status through to a second generation of offspring (Crudo et al. 2012). A potential insight into how glucocorticoid exposure could regulate metabolic and reproductive phenotypes across generations comes from the observation that synthetic glucocorticoid administration can alter gene expression in the hypothalamo–pituitary–adrenal (HPA) axis. Furthermore pituitary hormone levels are modified by this intervention through to a second generation of female offspring (Iqbal et al. 2012). Similar effects have been noted across species, in particular the sheep where HPA axis activity was up-regulated at baseline function, but displayed a blunted response to stimulation in the F2 generation (Long et al. 2013). Given the central role of the HPA axis in coordinating and facilitating female reproductive function, these studies demonstrate a likely central role for glucocorticoid exposure in early-life in transgenerational developmental programming of reproductive function.

#### Accelerated cellular ageing

The final major mechanism that is often postulated for developmental programming effects is accelerated cellular ageing. Rapid cellular ageing and decreased longevity have been postulated as key elements of developmental programming phenotypes since the earliest landmark studies in animal models were published (Jennings et al. 1999; Ozanne and Hales 2004). Rapid shortening of telomere length and telomerase alterations have been observed in many tissues in F1 offspring in response to suboptimal diets during the F0 pregnancy (Ng et al. 2014b; Tarry-Adkins et al. 2012). The same effect has been seen in human infants in response to maternal dietary alterations (Entringer et al. 2015). Tissues in which early decline in telomere length has been observed in animal models include not only key metabolic sites such as the pancreatic islets (Tarry-Adkins et al. 2009), but also reproductive tract tissues (Aiken et al. 2013). Evidence of accelerated telomere shortening in the somatic reproductive tract tissues of second-generation offspring in a maternal low-protein diet rat model (Aiken et al. 2015) demonstrates that this effect may be recapitulated in subsequent generations. The accelerated ageing effect of maternal diet on the reproductive tract of adult female offspring means that F1 age at second-generation breeding may be a significant influence on the extent to which developmental programming phenotypes are transmitted or re-propagated through subsequent generations (Aiken and Ozanne 2014).

Although a decline in telomere length of second-generation offspring has not been directly observed in other metabolic and reproductive tract tissues, developmental programming phenotypes are characterized also by early accumulation of oxidative and nitrosative stress, and a significant upregulation of anti-oxidant defense mechanisms (Ozanne 2014; Tarry-Adkins et al. 2012). This is in keeping with the paradigm that an age-related shift towards an oxidized redox state is a precursor to frank cellular damage from oxidative stress (Brewer 2010) in sensitive metabolically active tissues. In the F1 generation of rats exposed to maternal low-protein diet, evidence suggests that both male (Rodriguez-Gonzalez et al. 2014) and female (Aiken et al. 2013; Bernal et al. 2010) gonads have a rapid accumulation of oxidative stress and alteration of anti-oxidant defenses. In the male in particular, these changes are demonstrable in the gametes, which provides a potential mechanism for affecting the F2 generation.

## Discussion

There is a body of evidence from animal models that establishes the paradigm that metabolic and reproductive effects can be transmitted across generations in at least some maternal dietary models (Aiken et al. 2015; Benyshek et al. 2006; Blondeau et al. 2002; Burdge et al. 2011; Graus-Nunes et al. 2015; Radford et al. 2014). It must also be noted that other studies have sought such effects and been unable to detect any phenotype in generations beyond F1 (Benyshek et al. 2004; Chernoff et al. 2009). From the insights afforded by a few key studies within the field, evidence for several different mechanisms by which such effects may be propagated is gradually accumulating. After initial establishment of the adverse programming by direct effects of the F0 pregnancy on the F1 generation, further transmission via the paternal line is highly likely to occur via epigenetic modulation of the spermatozoan nucleus (Radford et al. 2014), as this is the main contribution of the F1 father to the phenotype of the F2 offspring. Via the maternal line, the possibilities for transmission are much more complex and include epigenetic modification not only of the gamete but also programming via the ooplasm, the reproductive tract environment or maternal physiology adapting poorly to the demands of pregnancy [reviewed in (Aiken and Ozanne 2014)]. There is much further scope for developing transgenerational animal models to disentangle these possibilities.

Determining whether such effects are truly present in human populations presents a variety of epidemiological challenges (Pembrey et al. 2014). These centre around the difficulties of accurately determining exposures, particularly maternal dietary exposures and of delineating outcomes, particularly reproductive outcomes. The long generation times in human cohorts, as well as the heterogeneity in experience that exists within populations makes the design and feasibility of such studies incredibly complex (Susser et al. 2012). Useful results thus far have emerged from the careful identification of historical cohorts in isolated populations where exposure is known to be relatively homogeneous across the population, for example during the Dutch hunger winter, when food rationing was strictly adhered to (Lumey et al. 2007), or the very isolated Overkalix population where there was little outside influence on food availability (Bygren et al. 2001). It seems likely that further insights could also be gleaned from careful use of such important ‘natural experiments’ either in isolated populations, those experiencing particular dietary challenges, or where unusual social structures are adhered to. This will be especially important with regard to reproductive outcomes, which present a particular difficulty in being influenced by choice rather than biological capacity in many populations. In the future, transgenerational effects dependent on maternal diet may become much more amenable to study in human populations, due to the proliferation of well-characterized prospective birth cohorts that have been set up in many countries. For example, a transgenerational effect of grand-maternal smoking on birthweight has been reported from the US Collaborative Perinatal Project (CPP), to which the grandmothers were recruited in the early 1960s (Misra et al. 2005). Within the next few decades, a future generation of data will be available from contemporary, well-characterized, birth cohorts—importantly including those with biobanks such as the ALSPAC cohort from the UK, which shows methylation differences with maternal bodyweight in the first generation of offspring (Sharp et al. 2015). Inevitably, while older cohorts such as those developed in the 1950s and 1960s [for example the National Child Development Study (NCDS) (Peckham 1973)] will become useful for studying transgenerational effects within a shorter time-frame, more modern cohorts are larger, better characterized and have more biological material available and stored in an appropriate way for epigenetic analysis [reviewed in (Pembrey et al. 2014)]. Some of the methodological issues with drawing conclusions from human cohorts regarding dietary intake may be addressed by other study designs, including transgenerational follow-up of nutritional intervention studies and the use of sibling controls (Donovan and Susser 2011). However, such cohorts represent a very long time-scale to obtain results, particularly as metabolic and fertility outcomes will be most evident well into the adult lives of grand-offspring.
